# The wealth gradient and the effect of COVID-19 restrictions on income loss, food insecurity and health care access in four sub-Saharan African geographies

**DOI:** 10.1371/journal.pone.0260823

**Published:** 2021-12-15

**Authors:** Elizabeth Gummerson, Carolina Cardona, Philip Anglewicz, Blake Zachary, Georges Guiella, Scott Radloff

**Affiliations:** 1 Department of Population Family and Reproductive Health, Johns Hopkins Bloomberg School of Public Health, Baltimore, Maryland, United States of America; 2 Department of Health Behavior and Society, Johns Hopkins Bloomberg School of Public Health, Baltimore, Maryland, United States of America; 3 L’Institut Superieur des Sciences de la Population (ISSP), L’Universite Joseph Ki-Zerbo de Ouagadougou, Burkina Faso; Kansas State University, UNITED STATES

## Abstract

**Introduction:**

While there has been considerable analysis of the health and economic effects of COVID-19 in the Global North, representative data on the distribution and depth of social and economic impacts in Africa has been more limited.

**Methods:**

We analyze household data collected prior to the COVID-19 pandemic and during the first wave of COVID in four African countries. We evaluate the short-term changes to household economic status and assess women’s access to health care during the first wave of COVID-19 in nationally representative samples of women aged 15–49 in Kenya and Burkina Faso, and in sub-nationally representative samples of women aged 15–49 in Kinshasa, Democratic Republic of Congo and Lagos, Nigeria. We examine prevalence and distribution of household income loss, food insecurity, and access to health care during the COVID-19 lockdowns across residence and pre-pandemic wealth categories. We then regress pre-pandemic individual and household sociodemographic characteristics on the three outcomes.

**Results:**

In three out of four samples, over 90% of women reported partial or complete loss of household income since the beginning of the coronavirus restrictions. Prevalence of food insecurity ranged from 17.0% (95% CI 13.6–20.9) to 39.8% (95% CI 36.0–43.7), and the majority of women in food insecure households reported increases in food insecurity during the COVID-19 restriction period. In contrast, we did not find significant barriers to accessing health care during COVID restrictions. Between 78·3% and 94·0% of women who needed health care were able successfully access it. When we examined pre-pandemic sociodemographic correlates of the outcomes, we found that the income shock of COVID-19 was substantial and distributed similarly across wealth groups, but food insecurity was concentrated among poorer households. Contrary to a-priori expectations, we find little evidence of women experiencing barriers to health care, but there is significant need for food support.

## Introduction

Starting in the spring of 2020, policy makers across the globe were faced with the unenviable task of charting a path between limiting catastrophic loss of life from the COVID-19 pandemic, while also trying to avoid an economic downward spiral that could, in itself, lead to large scale human suffering. Prior to the advent of effective vaccines, the most effective tools to reduce the spread of the virus were policies to reduce the mobility and contact between human populations through closures of schools, marketplaces, churches and public gatherings. In many countries, lockdowns additionally kept citizens in their homes unless they had explicit permission to leave. While these tactics likely helped contain the morbidity and mortality from pandemic, they were also associated with a host of secondary effects such as loss of schooling, loss of income, reduced access to health care, supply chain disruptions, increased prices for food and goods, and increased food insecurity [1–3].

As COVID-19 was concentrated in high income countries (HIC) during the early phase of the pandemic, much of the early population-based data on both primary and secondary effects of COVID-19 also came from HICs. Early analysis demonstrated that the social and economic consequences of COVID-19 disproportionately impacted low wage manual workers, women and the poor [4, 5]. Many predicted similar patterns in lower-income countries, only with more severe consequences, as a result of widespread poverty, limited social safety net programs, and large informal sectors that are particularly vulnerable to lockdown measures [6–8].

As the global pandemic enters a third wave at the time of this writing, social scientists are still gathering data on resilience of health and social infrastructure, economic systems, and individual households to both the virus and accompanying shutdowns. However, one emergent theme is that the trajectory of the COVID-19 pandemic in Africa does not necessarily mimic the trajectory of the pandemic in the Global North [9, 10]. Some feared that Africa might be particularly vulnerable to COVID-19 mortality and morbidity as a result of weaker health infrastructure and endemic challenges such as HIV, malaria and malnutrition, creating large immunocompromised populations [11–13]. But many African countries have been spared the scale of mortality that was seen in China, Europe and North America during the first wave of the pandemic [1, 10, 14, 15]. Some of this difference is attributable to more limited testing, resulting in COVID-19 deaths going underreported [16–19], and the pandemic in South Africa has been a well-documented exception [20]. Still, many African countries appear to have benefited from factors such as quick intervention on the part of governments, a comparatively young population, warmer climate, widespread adherence to infection control measures, and higher barriers to movement for many people- resulting in less excess mortality during the first wave than was seen elsewhere [9, 10, 21–23].

In contrast, the secondary social and economic effects appear to be significant for the African continent [1, 3, 24]. United Nations (UN) estimates place GDP contraction in African countries between 1.4% and 7.8%, and the World Bank estimates that the pandemic pushed over 40 million Africans into extreme poverty [3, 25]. Furthermore, according to the African Center for Strategic Studies, food insecurity in Africa increased in 2020 by 60% over the prior year [26]. The depth and persistence of these secondary effects are heterogeneous between countries and even within communities, and researchers have not found entirely consistent patterns of vulnerability. Using the World Bank’s Living Standards Measurement Surveys (LSMS) in four countries (Nigeria, Malawi, Ethiopia and Uganda), Jospehson et al (2020) found no evidence of differential levels of income loss between rural and urban populations, but found that households that were poorer at baseline were more likely to suffer food insecurity and disrupted access to staple foods [27]. Another study in Addis Ababa also found that poorer households were more likely to report both income loss and food insecurity during the COVID-19 pandemic [28]. In contrast, a study in Malawi found that although food security was worse in rural areas prior to COVID-19, food security deteriorated more in urban areas during the first wave of the pandemic, erasing the prior urban advantage [29]. Similarly, a study in rural communities in Uganda found that the negative economic effects were actually larger among households that were wealthier at baseline [30]. An analysis in South Africa found that the initial income effects were similar in urban and rural areas, but that urban areas experienced income and employment recovery at a higher rate, widening urban-rural inequality [31].

Our study adds to the evidence base on the secondary effects of COVID in sub-Saharan Africa. We use longitudinal representative household data, with baseline data from before the COVID-19 pandemic (collected between November 2019 and February 2020), combined with follow-up data collected during the first set of COVID-19 restrictions (May to August 2020) in four African countries to evaluate the depth and breadth of the short term social and economic changes during the first wave of COVID-19. Our data comes from four representative panel surveys of women ages 15–49 and their households, including data from two national samples in Burkina Faso and Kenya, and two urban samples in Kinshasa, DRC and Lagos, Nigeria. We focus on three socioeconomic outcomes: 1) household income loss, 2) food insecurity, and 3) access to health care. We then look at the difference in these outcomes across urban and rural households (in the two national samples), and stratified by pre-pandemic wealth categories (in all four samples). Finally, we use logistic and multinomial logistic regression techniques to examine both individual and household level correlates of the three outcomes.

## Materials and methods

This study uses data from the Performance Monitoring for Action (PMA) platform, which follows nationally, or sub-nationally representative cohorts of women aged 15–49 to track key reproductive health indicators in nine geographies across sub-Saharan Africa and India. PMA uses a multi-stage stratified cluster design, starting with the random selection of geographical enumeration areas (EAs), comprised of approximately 200 households, based on the relevant national census, followed by the random selection of 35 households from each area. All women ages 15–49 living in the selected households and who provide informed consent are included in the panels.

This analysis uses data from two PMA nationally representative panels of women aged 15–49 in Burkina Faso and Kenya, and two sub-nationally representative urban panels of women in Kinshasa, DRC and Lagos, Nigeria. The PMA project collected in-person baseline data on the women and their households in these four geographies between November 2019 and February 2020, prior to any COVID-19 cases being recorded in these countries. When the COVID-19 pandemic emerged, the PMA interviewers re-contacted the panel participants in these geographies to administer a phone-based follow up survey focusing on the impact of COVID-19. The phone survey collected information on women’s knowledge, awareness, and practices related to COVID-19; the economic consequences of COVID-19; changes in fertility and reproductive health behaviors; and COVID-related barriers to health services. The COVID phone questionnaire is attached as S1 Appendix. The phone surveys were collected between 28^th^ of May and 14^th^ of August 2020.

In all four geographies, national governments had implemented restrictions on movement and gatherings, including closures of schools and business in the two to three months prior to the interviews. In Kenya, national policy restricted travel and social gatherings, and mandated work from home starting on March 15^th^, 2020. By March 25^th^ an additional national curfew was implemented, and all learning institutions had been closed. The Kenyan government began lifting the first-round restrictions in early July of 2020. Interviews took place between June 1^st^ and 17^th^, approximately 10 weeks into the restrictions. In Burkina Faso, schools were closed on March 16^th^ and restrictions on gatherings over 50 people and travel and a curfew were put in place March 20^th^. Schools reopened on May 15^th^ and curfew was lifted on June 5^th^. Interviews took place June 25^th^–July 10^th^, shortly after the first round of restrictions had been lifted. In Lagos, schools were closed in mid-March and full restrictions, closing business, prohibiting gatherings and limiting movements, were in place from March 30^th^ to May 5^th^, when a phased lifting of restrictions began. Schools resumed June 30^th^ and curfews and travel restrictions remained in place into late July. PMA interviews took place between July 15^th^ and August 14^th^. In Kinshasa a series of lockdown measures were announced between March 19^th^ and 24^th^, closing restaurants, business, schools, transport companies, and restricting movement outside of homes. These restrictions stayed in place until August 15, 2020, when schools and some business reopened. PMA phone interviews took place between May 28^th^ and June 19^th^, beginning approximately 9 weeks into the restriction period.

We used data from the baseline surveys conducted among 6,590 women in Burkina Faso, 9,477 in Kenya, 2,611 women in Kinshasa, and 1,456 women in Lagos to provide information on the demographic characteristics of women and their households prior to the COVID-19 pandemic. The percentages of women from baseline survey that owned phones were 83.3% in Lagos, 57.6% in Burkina Faso, 68.1% in Kinshasa and 72.3% in Kenya. The response rates among eligible phone-owning women for the COVID-Phone survey were 75.2% in Burkina Faso, 81.7% in Lagos, 74.7% in Kinshasa, and 93.9% in Kenya. A total of 3,522 women in Burkina Faso, 5,982 women in Kenya, 1,286 women in Kinshasa and 957 women in Lagos completed the COVID-19 survey.

The data from the COVID-19 phone survey were weighted to be representative of women and households in each national and sub-national sample. The weighting procedures adjusted for sampling and the likelihood of attrition from the baseline survey to the COVID-19 survey. We further applied inverse probability weights to account for differences in phone ownership based on characteristics of phone owners and non-phone owners in the baseline survey. A detailed description of the PMA weighting, protocol, and other procedures are available on the PMA website (pmadata.org) [32]. We restricted our analysis to women who answered both the baseline and COVID-19 surveys.

The PMA surveys are conducted in partnership with The Bloomberg School of Public Health at Johns Hopkins University; L’Institut Superieur des Sciences de la Population, L’Universite Joseph Ki-Zerbo de Ouagadougou; Ecole de Sante Publique Universite de Kinshasa; International Center for Reproductive Health, Kenya; Center for Research Evaluation and Resources Development, Nigeria; and University of Ibadan, Nigeria. Researchers received ethical approval for conducting the COVID-19 surveys from institutional review boards in each country including the Comité d’Ethique Institutionnel Pour La Recherche en Santé (Burkina Faso—No. A14-2020); Kenyatta National Hospital-University of Nairobi Ethics Research Committee (Kenya—No. P241/04/2020); The Lagos State University Teaching Hopspital Health Research Ethics Committee (LREC /06/10/1276); the Comité d’Ethique Ecole de Sante Publique Universite de Kinshasa (ESP/CE/78/2020); and the Johns Hopkins Bloomberg School of Public Health (IRB No. 12407). All interviewed respondents provided electronic informed consent. Married minors (15–17) were treated as adults in all countries. Unmarried minors (15–17) were treated as adults in Burkina Faso and DRC; in all other countries, consent was granted by the parent and assent given by the respondent. All consent procedures were approved by the relevant ethical review board. Written consent was obtained in Kenya. In the other three geographies, verbal consent was obtained (and documented electronically) due to low levels of literacy in the survey population. All analyses performed here were performed on an anonymized dataset.

### Outcomes and empirical approach

To measure the changes in outcomes due to COVID-19 at the individual level, we followed a simple analytic framework aiming to capture women’s potential outcomes in two alternate states of the world, one with COVID-19 and one without. As we aren’t able to directly observe both states simultaneously, we asked women for their subjective assessments of how household income and food security had changed since the beginning of the COVID-19 restrictions, and whether they had experienced difficulty accessing health care during the restrictions. In all geographies the survey was conducted during or just after the first wave of COVID-related restrictions. So respondents were assessing a single round of restrictions, although the length of the full restriction period varied slightly between geographies, ranging from 9 to 11 weeks. We measured household income loss via a question on whether the respondent’s household had experienced complete, partial, or no loss of household income since the onset of the COVID-19 restrictions. We measured food insecurity with a question on whether any adult in the woman’s household had gone 24 hours without eating during the COVID-19 restrictions because there was no food in the household. We did not have a pre-pandemic measure of food insecurity in the survey households, but among households reporting food insecurity, respondents reported on whether food insecurity had increased since the start of the coronavirus restrictions. Finally, we assessed access to health care by first asking if the respondent needed to access health care services during the COVID-19 restriction period, and then if she had been able to successfully access the needed care.

We examined the overall levels of our three primary outcome measures in each country and by urban residence (for the national samples). We then stratified our analysis by country and wealth tertile to analyze outcome patterns by pre-pandemic household wealth. Pre-pandemic wealth tertile was calculated using a factor score constructed from a principal components analysis of household characteristics, including assets and household materials, and divided into tertiles following Filmer & Pritchett (2001) [33]. One particular strength of the data is that all of our baseline sociodemographic characteristics were measured prior to COVID-19, so our household wealth scores were not impacted by the effects of the pandemic. Finally, we conducted regression analyses to identify sociodemographic correlates of our three outcomes in each national and sub-national sample. In the regression analyses we regressed both individual and household characteristics, measured before the pandemic (including marital status, age, parity, education, employment status, household wealth, household size and urban residence), on the three outcomes measured during the COVID-19 phone survey. For the categorical outcome household income loss, we conducted multinomial logistic regression to examine the correlates of complete and partial household income loss. We also tested an ordered probit regression for household income loss; however, a Brant test of the proportional odds assumption did not hold. As such, we present the outcomes as multinomial outcomes rather than an ordered probit. For the two binary outcomes, food insecurity and health care access, we performed binomial logistic regression. All analyses were conducted using Stata16.1 (College Station, TX). Analysis was performed on the full set of women who answered the COVID-19 survey, outliers were not trimmed. All regression analyses included controls for country and subnational region (where applicable) and are weighted, as described above, to account for the complex survey design and attrition. Regression standard errors are clustered at the EA level.

## Results and discussion

### Results

**Fig 1:**
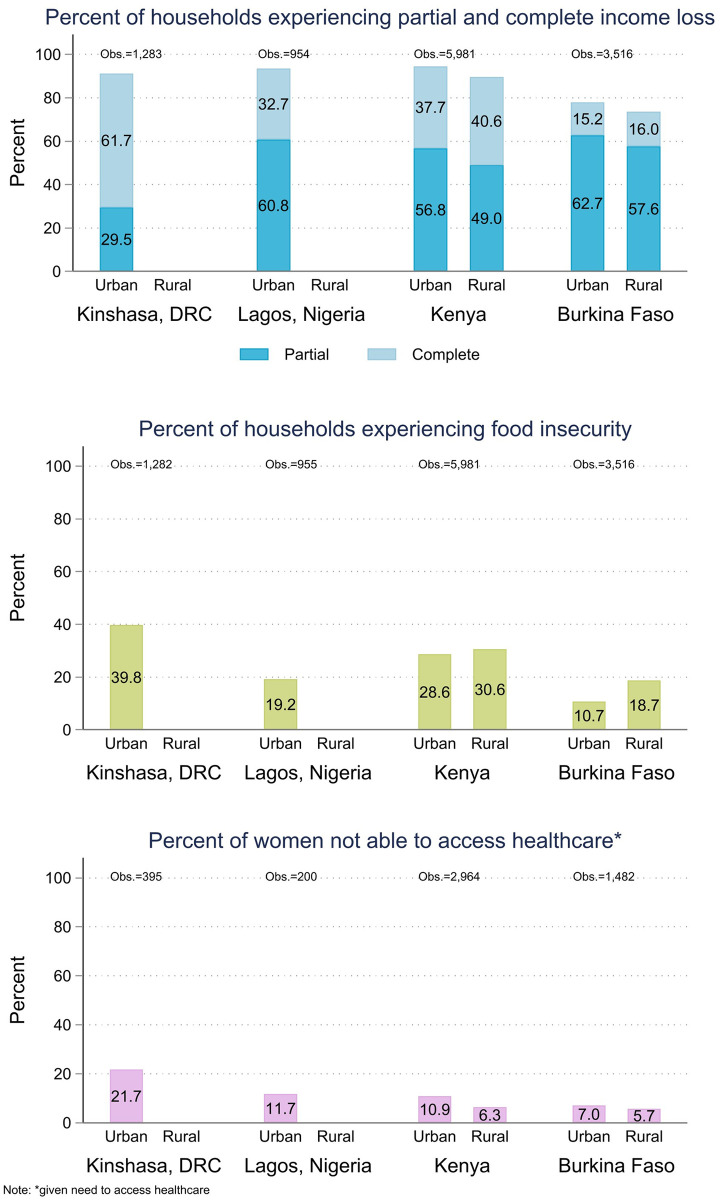
Household income loss, food insecurity, and access to health care by residence.

The overall levels of our three outcome variables are shown in Fig 1 stratified by country and urban residence where applicable. Country specific percentages are shown for each of the outcomes and the pre-pandemic socioeconomic characteristics in Table 1. In all four geographies, the respondents reported high levels of income loss. Burkina Faso had the lowest percentage of women reporting complete or partial loss of household income at 76.8% (95% CI, 69.3–79.2). In all other geographies, more than 90% of women reported either complete or partial loss of household income since the beginning of the coronavirus restrictions. Similarly, respondents reported high levels of food insecurity, with 39.8% (95% CI, 36.0–43.7) of women in Kinshasa and 30.1% (95% CI, 27.4–32.9) of women in Kenya reporting that an adult in their household had gone at least 24 hours without any food due to food scarcity. In Burkina Faso and Lagos, Nigeria, reported levels of severe food insecurity were lower, 17.0% (95% CI, 13.6–20.9) and 19.2% (95% CI, 16.1–22.8) respectively, but still high compared to national estimates from prior years. For comparison, the Food and Agricultural Organization of the United Nations (FAO) estimates prevalence of severe food insecurity between 2017 and 2019 to be 12.6% in Burkina Faso, 23.3% in Kenya. Similar comparisons for the urban areas of Kinshasa and Lagos are not published, but the FAO national estimate for Nigeria was 14.8% (an estimate for DRC in the same time period was not available) [34]. In Burkina Faso, data collection took place shortly after a harvest season, which may have provided some protection against food insecurity-particularly for rural areas. In Kenya, data collection aligned with the start of a lean season in the agricultural year for much of the country, and Kenyan farmers were additionally facing once generational locust swarms, which likely impacted overall levels of food insecurity [27, 35].

**Table 1:** Characteristics of women, aged 15–49, in Kenya, Burkina Faso, Lagos, Nigeria and Kinshasa, DRC who completed the COVID-19 follow up survey.

	Kenya		Burkina Faso		Lagos, Nigeria		Kinshasa, DRC	
***Outcome Variables- Measured during COVID surveys***	**%**	**N**	**%**	**N**	**%**	**N**	**%**	**N**
**Household Income Loss**								
No income loss	8.0	476	23.2	814	5.97	57	8.3	107
Partial Income Loss	52.4	3135	61.1	2149	60.0	572	33.7	432
Complete Income Loss	39.6	2370	15.7	553	34.1	325	58.0	744
**Food Insecurity**								
Household experienced food insecurity	30.1	1738	19.2	426	17.0	183	39.8	472
Increase in food insecurity compared to pre-COVID-19*	72.4	1261	60.3	264	73.6	134	71.7	325
**Access to Health Care**								
Needed Health Services during COVID-19 restrictions	48.5	2964	43.1	1483	20.4	200	31.3	395
Received Health Services during COVID-19 restrictions**	92.5	2705	88.3	1371	94	176	78.3	319
***Individual characteristic variables*, *measured pre-pandemic***								
**Marital status**								
In union	60.2	3598	75.3	2653	61.7	590	45.3	582
Not in union	39.8	2382	24.7	868	38.3	366	54.7	703
**Parity**								
0	26.7	1598	28.1	988	34.6	331	43.4	559
1	17.9	1073	14.9	526	11.1	106	14.2	182
2–3	29.5	1766	24.2	851	34.0	325	23.1	297
4+	25.8	1545	32.8	1155	20.3	194	19.3	248
**Age group**								
15–24	41.0	2452	45.3	1595	27.8	266	43.5	560
25–34	31.5	1882	28.9	1017	33.3	319	28.5	366
35–49	27.6	1648	25.8	910	38.9	372	28.0	360
**Education**								
None/Primary	52.8	3161	77.6	2732	11.4	109	8.7	112
Post-Primary/Secondary	35.6	2128	20.7	729	51.6	493	72.3	930
Tertiary/College	11.6	693	1.7	59	37.0	354	19.0	244
**Work status and remuneration**								
Unemployed	42.1	2519	45.6	1607	17.8	171	47.2	607
Cash	37.8	2259	33.4	1175	74.7	715	45.5	584
Cash/In-kind/Not paid	20.1	1202	21.0	739	7.5	72	7.3	94
***Household characteristic variables-measured pre-pandemic***								
**Members in the household**								
1–3	23.5	1404	17.1	601	22.5	216	12.2	158
4–6	50.3	3011	31.8	1119	64.5	617	45.2	581
7+	26.2	1567	51.2	1802	13.0	125	42.6	547
**Wealth**								
Lowest	39.4	2359	60.8	2141	35.3	338	28.8	370
Middle	31.4	1877	25.4	894	32.2	308	33.8	434
Highest	29.2	1747	13.8	487	32.5	311	37.4	481
**Rural/Urban**								
Urban	38.3	2288	25.3	890	100.0	957	100.0	1286
Rural	61.8	3694	74.7	2632	na	na	na	na

Note:

*Conditional on reporting household food insecurity

**Conditional upon reporting needing to access health care.

Descriptive statistics are weighted for the inverse probability of participating in both survey rounds.

The percentage of women reporting needing health care during the COVID-19 restrictions ranged from 20.4% (95% CI, 17.6–23.5) in Lagos to 48.5% (95% CI, 46.1–50.9) in Kenya. The vast majority of women who reported needing health care services during the restrictions also reported that they were able to access the health care they needed. Kinshasa had the lowest proportion of women reporting successful health care access with 78.3% (95% CI, 68.8–85.5). In the other three geographies the percentages successfully accessing health care ranged between 88.4% (95% CI, 82.7–92.2) of women in Lagos and 94.0% (95% CI, 91.5–95.8) in Burkina Faso.

Fig 1 shows the three primary outcomes stratified by country and rural residence. In the two countries where the samples were national, there was no discernable difference in overall levels of income loss or access to health care between urban and rural households. Similarly, we found no statistically significant differences in the risk of food insecurity in urban versus rural regions of Kenya. However, women in rural regions in Burkina Faso had a comparatively elevated risk of household food insecurity with 18.6% (95% CI, 14.6–23.7) reporting food insecurity compared to 10.7% (95% CI, 9.1–12.5) in urban regions.

**Fig 2:**
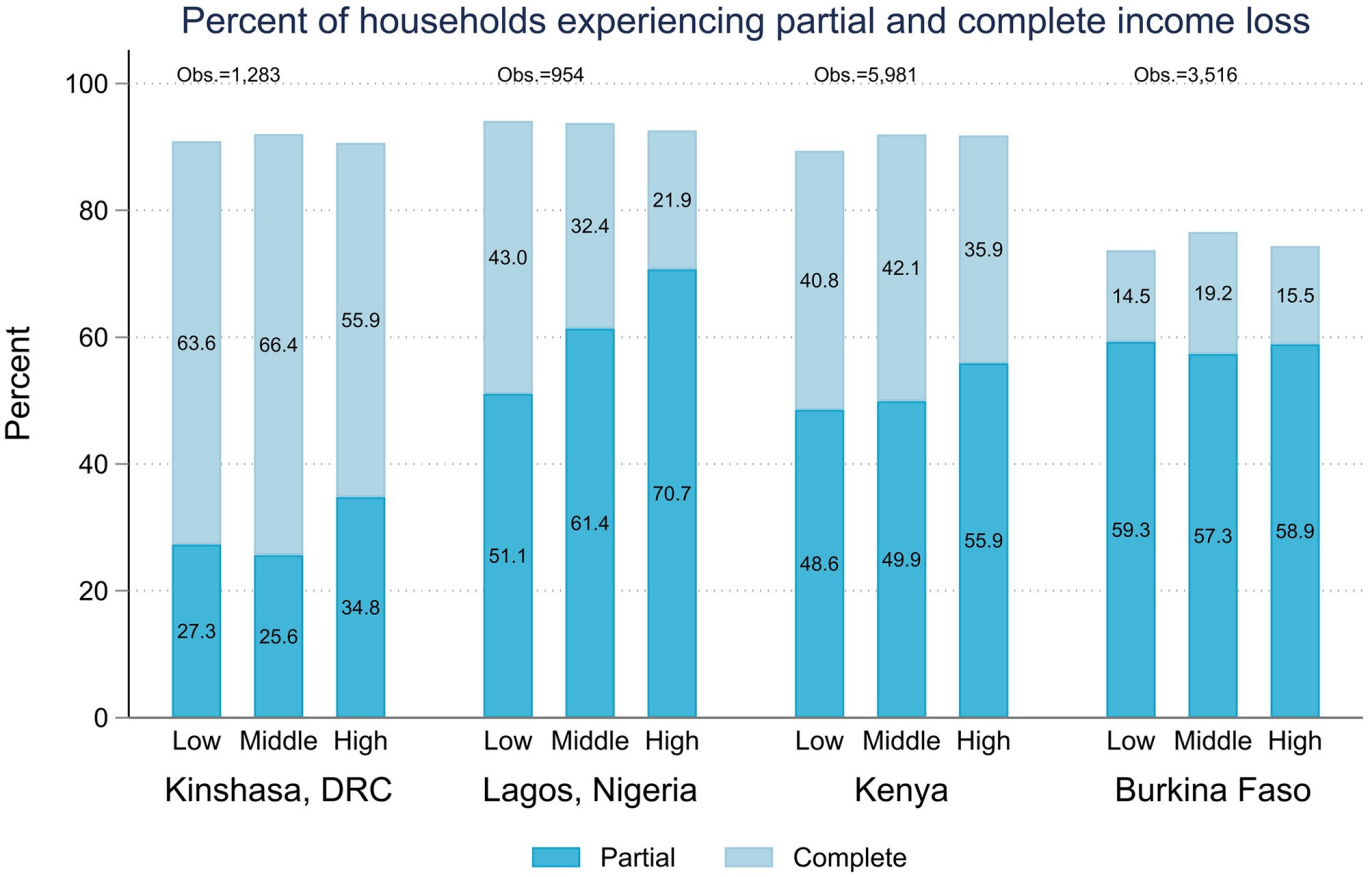
Prevalence of partial and complete household income loss during COVID-19 restrictions across pre-pandemic wealth tertiles.

**Fig 3:**
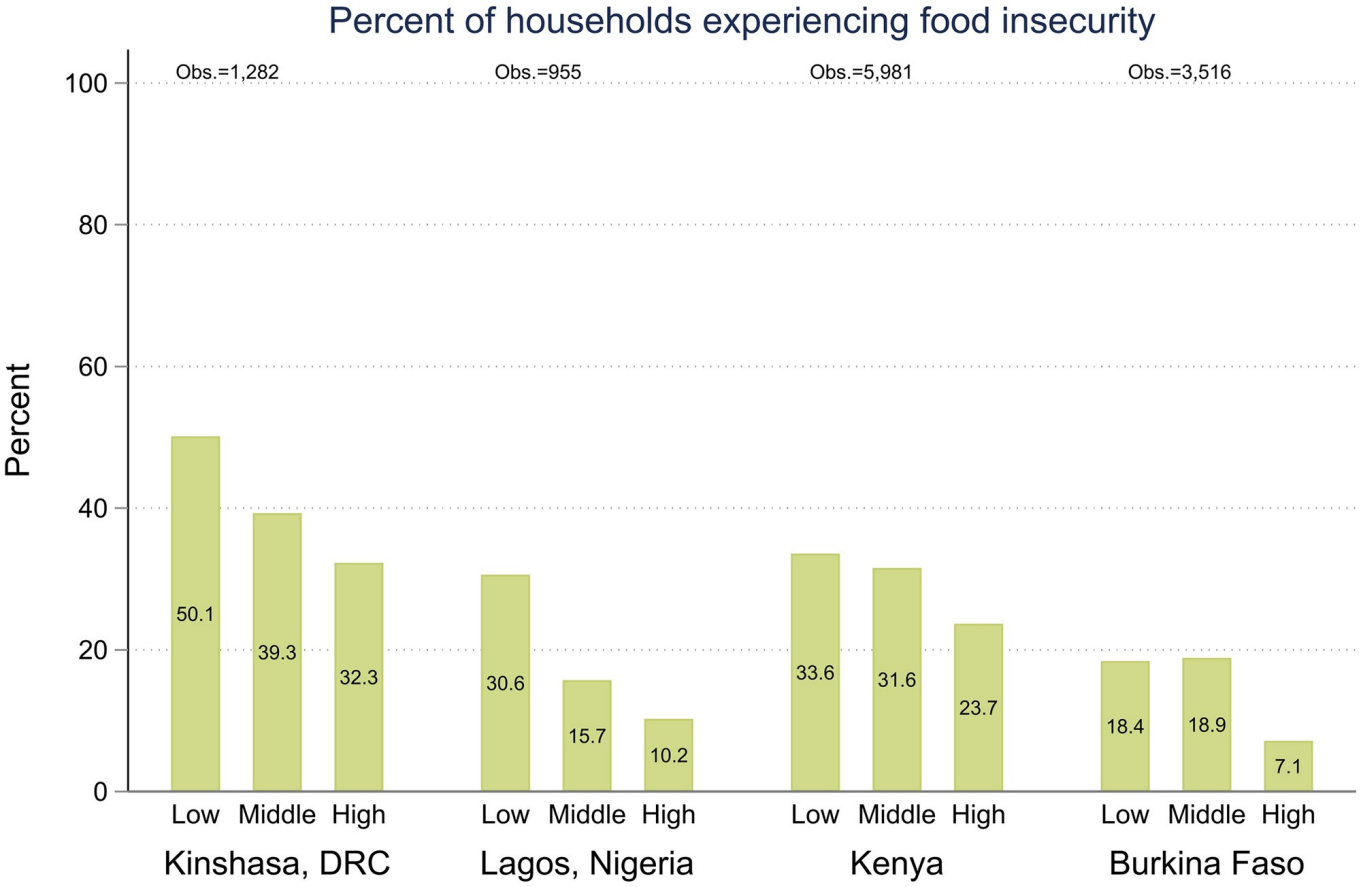
Prevalence of food insecurity during COVID-19 restrictions across pre-pandemic wealth tertiles.

**Fig 4:**
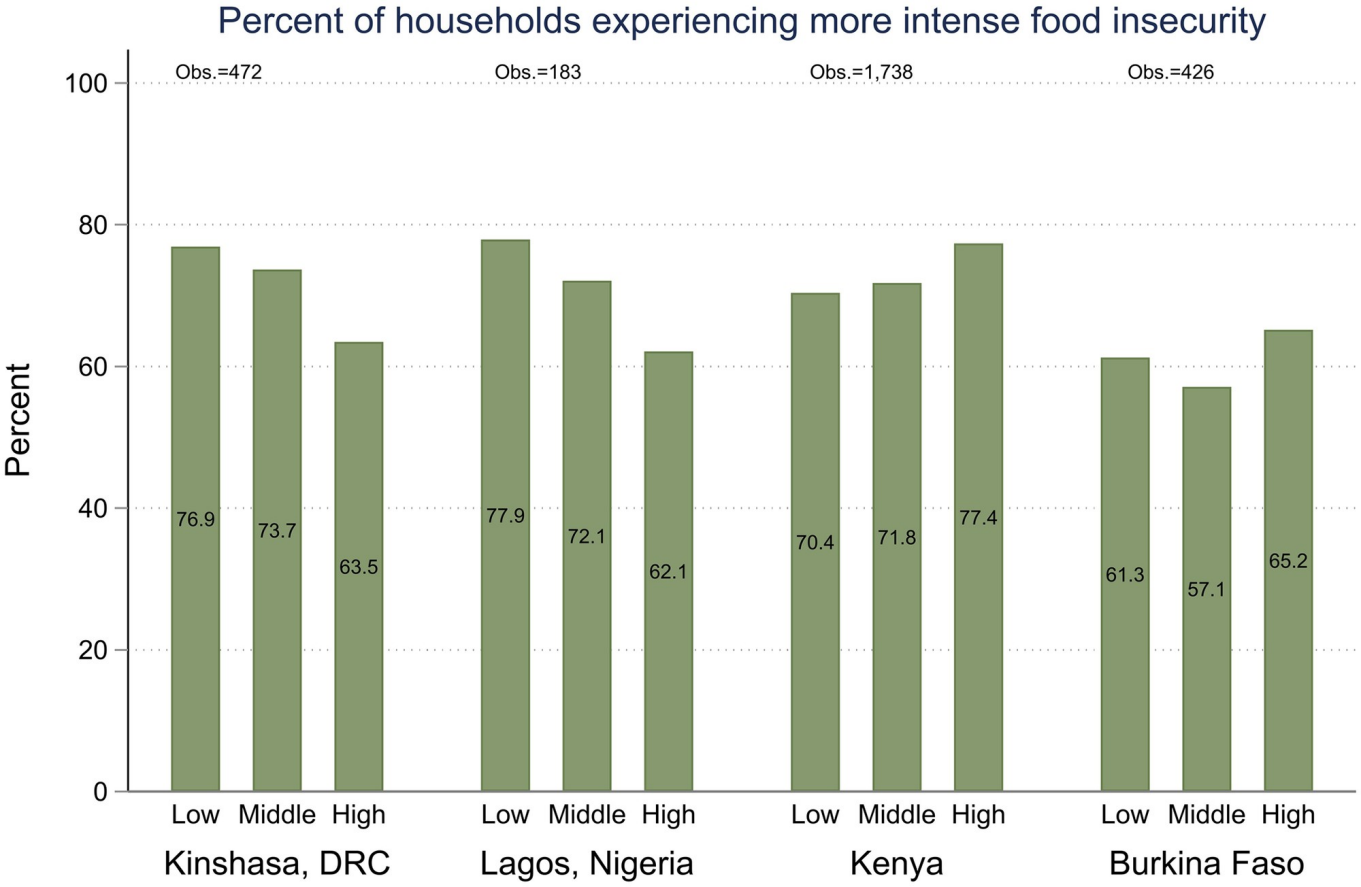
Prevalence of increased food insecurity during COVID-19 restrictions across pre-pandemic wealth tertile.

**Fig 5:**
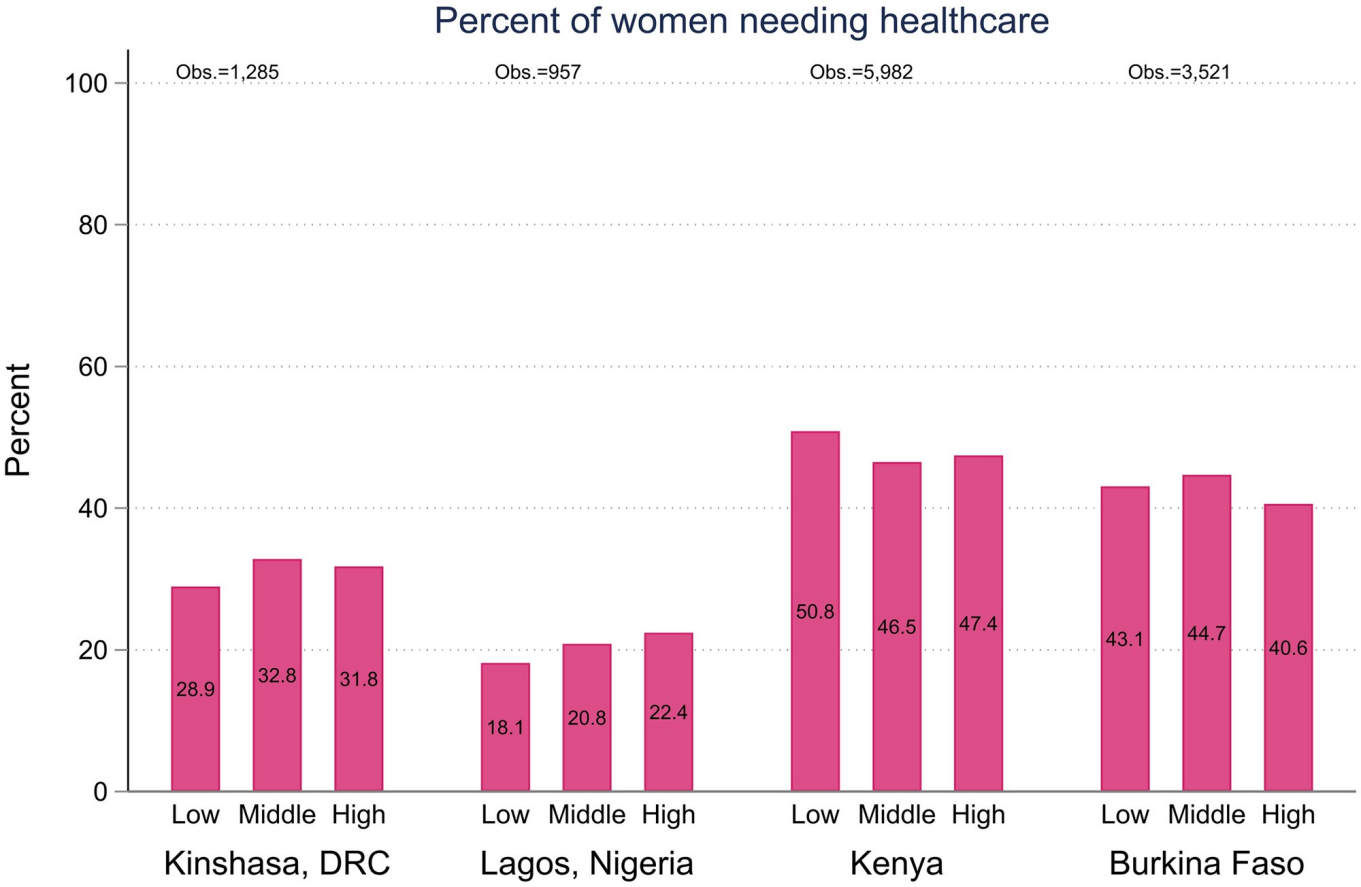
Percent of women, aged 15–49, reporting needing health care during COVID-19 restriction.

**Fig 6:**
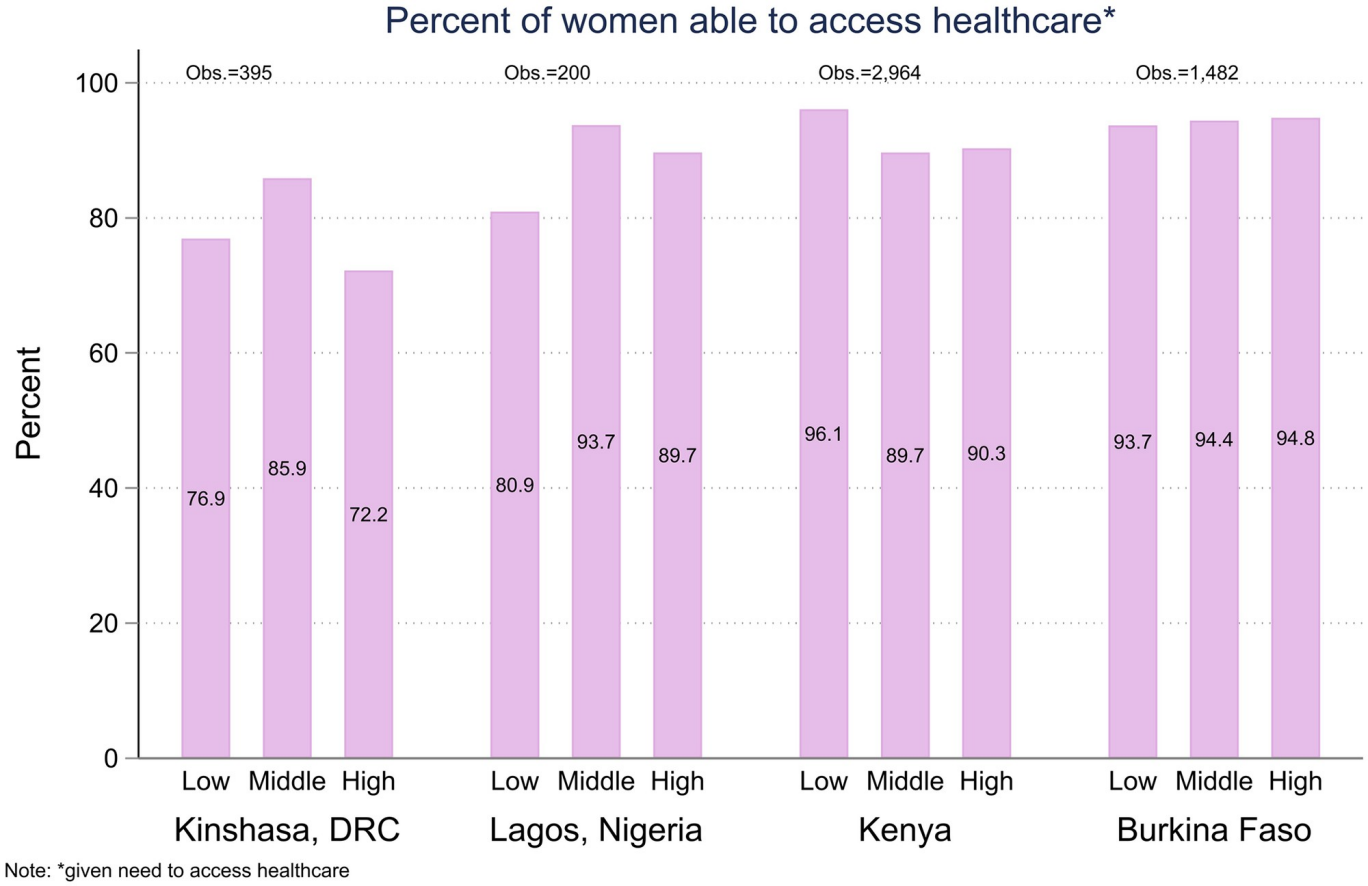
Percent of women needing health care, aged 15–49, who report success accessing health care.

Figs 2–6 show the wealth gradient in each country for each of the outcome variables listed in Table 1. For household income loss (Fig 2) we found a wealth gradient only for the Lagos sample, where women across the three wealth categories reported similar levels of overall household income loss, but those in the highest wealth tertile were more likely to report partial income loss (70.7%, 95% CI, 63.6–76.9%) whereas a higher percentage of women in the lowest income tertile reported complete income loss (43.0%, 95% CI, 35.6–50.8%). In the other three geographies, there was little discernible pattern in the experience of income shock based on the household’s baseline wealth, and differences between wealth tertiles were not statistically significant.

In contrast, the experience of food insecurity (Fig 3) showed a clear wealth gradient. Across all four geographies, the percentage of women reporting food insecurity reduced as the household’s wealth category increased from the lowest to the highest tertile. In Kinshasa the differences between wealth tertiles were non-significant. In, Kenya and Burkina Faso, there was no statistically significant difference between the two-lower wealth tertiles, but those in the highest wealth tertile were less likely to report food insecurity at 23.7% (95% CI, 20.8–26.8) and 7.1% (95% CI, 5.5–9.3), respectively. In Lagos, those in the lowest wealth category were significantly more likely to be food insecure at 30.6% (95% CI, 24.7–37.2), while the differences between the middle and highest tertile were non-significant.

The respondents additionally reported on their perception of whether their household’s experience of food insecurity had worsened since the start of the pandemic restrictions. In all geographies the majority of women in food insecure households reported that food insecurity had worsened since the start of the pandemic restrictions. Fig 4 shows the prevalence of women who reported increases in food insecurity by pre-pandemic wealth category. Although the differences are not statistically significant, in both urban samples the women in the lowest income category reported increases in food insecurity at a higher prevalence. This aligns with early reporting that food security of the urban poor was uniquely impacted by the COVID-19 lockdowns [36, 37]. In the two national samples the pattern is reversed. Women from households in the highest wealth category report the highest prevalence of increases in food insecurity.

Figs 5 and 6 show similar analysis of women’s reports of needing access to health care (Fig 5) and success in accessing health care since the beginning of the Coronavirus restrictions (Fig 6). At this early point in the pandemic, we did not find a statistically significant relationship between pre-covid wealth and needing health care, nor did we detect a wealth gradient in access to health care among those who need it. The differences between wealth quintiles were statistically non-significant in all four geographies. Kinshasa had comparatively higher levels of women reporting barriers to accessing care, but it was not systematically associated with household wealth. Women in the lowest wealth tertile in Lagos reported barriers to accessing care in slightly higher numbers, but the difference was not statistically significant.

Finally, Tables 2–4 present the multinomial and binomial logistic regression results. The tables show the relative risk ratios (RRR) for partial or complete household income loss and the odds ratios (OR) for severe food insecurity and health care access and their association with the pre-pandemic sociodemographic characteristics of the women and their households. Several patterns emerge across the analyses. For income loss (Table 2) women with children were at higher risk than women without children of experiencing household income loss in Kinshasa, Lagos and Kenya, controlling for all other sociodemographic variables. In Burkina Faso, the difference was non-significant for women with one child, but women with more than 2 children faced higher rates of partial income loss. Similarly, women with children reported more household food insecurity than women without children in all the samples, even controlling for potential confounders such as age, education, household size, and wealth.(Table 3) In the Kenya sample the association was significant for all levels of parity. In Lagos, it was significant for women with parity of 1 or 2–3 children, while in Kinshasa it was significant only for women with parity of 2–3 children and in Burkina Faso it was significant only for women with parity of 1.

**Table 2:** Individual and household factors associated with household income loss in Kenya, Burkina Faso, Lagos, Nigeria and Kinshasa, DRC [multinomial logistic regression].

	Kenya		Burkina Faso		Lagos, Nigeria		Kinshasa, DRC	
	Relative Risk Ratio [Standard Error]							
	Complete Household Income Loss	Partial Household Income Loss	Complete Household Income Loss	Partial Household Income Loss	Complete Household Income Loss	Partial Household Income Loss	Complete Household Income Loss	Partial Household Income Loss
**Marital status** *(ref*: *Married/In union)*								
Not Married/In Union	1.02	0.85	0.95	1.46	3.34**	1.89	1.26	1.02
	[0.21]	[0.17]	[0.43]	[0.70]	[1.69]	[0.96]	[0.50]	[0.41]
**Parity** *(ref*: *nulliparous)*								
1	1.80***	0.97	1.5	1.89	5.32***	2.6	2.65*	1.93
	[0.38]	[0.22]	[0.62]	[0.79]	[3.40]	[1.78]	[1.49]	[0.92]
2–3	2.98***	1.64**	0.69	1.783*	8.92***	5.257***	1.44	1.07
	[0.72]	[0.38]	[0.29]	[0.57]	[4.37]	[2.62]	[0.76]	[0.56]
4+	5.90***	2.90***	1.24	2.07**	5.57***	2.93*	2.04	1.04
	[1.81]	[0.92]	[0.58]	[0.71]	[3.44]	[1.81]	[1.22]	[0.64]
**Age group** *(ref*: *15–24)*								
25–34	1.41**	1.12	2.28**	1.08	1.21	0.57	3.43**	2.08
	[0.23]	[0.18]	[0.79]	[0.30]	[0.62]	[0.26]	[1.81]	[1.04]
35–49	1.53**	1.23	1.77	0.96	1.40	0.81	2.19	2.02
	[0.32]	[0.24]	[0.66]	[0.35]	[0.88]	[0.49]	[1.27]	[1.17]
**Education** *(ref*: *none/primary)*								
Post-Primary/Secondary	1.43*	1.42*	0.53**	0.70	1.02	1.00	1.16	1.58
	[0.26]	[0.26]	[0.16]	[0.20]	[0.70]	[0.625]	[0.75]	[1.02]
Tertiary/College	1.32	1.97***	0.22***	0.75	0.91	0.95	1.29	3.28*
	[0.30]	[0.40]	[0.08]	[0.22]	[0.64]	[0.68]	[0.85]	[2.17]
**Work status and remuneration** *(ref*: *not employed)*								
Paid work	1.19	1.35	1.00	1.03	0.86	1.22	1.27	1.13
	[0.23]	[0.25]	[0.30]	[0.20]	[0.35]	[0.50]	[0.35]	[0.34]
Informal in-kind or cash paid	1.11	1.14	2.01**	1.07	0.40	0.59	1.20	1.37
	[0.36]	[0.35]	[0.69]	[0.26]	[0.23]	[0.37]	[0.52]	[0.49]
**Members in the household** *(ref*: *1–3)*								
4–6	0.74	0.77	0.42**	0.77	0.47**	0.48**	1.24	1.37
	[0.14]	[0.15]	[0.18]	[0.24]	[0.18]	[0.16]	[0.54]	[0.52]
7+	0.61**	0.68*	0.52*	1.31	0.44	0.50	0.94	0.97
	[0.13]	[0.146]	[0.186]	[0.394]	[0.242]	[0.266]	[0.419]	[0.430]
**Household wealth tertile** *(ref*: *lowest)*								
Middle	1.03	1.06	1.45	1.16	0.67	1.12	1.26	1.00
	[0.23]	[0.23]	[0.39]	[0.32]	[0.29]	[0.54]	[0.45]	[0.36]
Highest	0.73	0.89	1.24	0.85	0.43**	1.16	0.90	0.97
	[0.18]	[0.20]	[0.39]	[0.25]	[0.18]	[0.50]	[0.27]	[0.34]
**Residence** *(ref*: *urban)*								
Rural residence	0.44***	0.44***	0.79	0.56**				
	[0.13]	[0.11]	[0.265]	[0.133]				
**Constant**	5.10***	7.87***	0.06***	1.18	2.68	6.85**	1.96	1.06
	[3.01]	[4.28]	[0.06]	[0.53]	[2.09]	[5.12]	[1.48]	[0.81]
**Observations**	5,978	5,978	3,510	3,510	952	952	1,282	1,282

Notes-; Reporting relative risk ratios and bolded values are statistically significant at *** p<0.01, ** p<0.05, * p<0.1; robust standard errors clustered at EA level and reported in brackets; regressions for Burkina Faso and Kenya include regional fixed effects; control variables measured at baseline (approximately 6 months prior to COVID survey); all regressions use inverse probability survey weights to account for attrition between rounds and phone ownership.

**Table 3:** Individual and household factors associated with household food insecurity in Kenya, Burkina Faso, Lagos, Nigeria, and Kinshasa, DRC [Logistic regression].

	Kenya	Burkina Faso	Lagos, Nigeria	Kinshasa, DRC
	Odds Ratio [Standard Error]			
**Marital status** *(ref*: *Married/In union)*				
Not Married/In Union	1.24**	0.77	1.60*	1.19
	[0.12]	[0.32]	[0.42]	[0.27]
**Parity** *(ref*: *nulliparous)*				
1	1.54**	2.18*	2.62***	1.10
	[0.26]	[1.00]	[0.96]	[0.21]
2–3	1.87***	1.30	2.04*	1.54*
	[0.36]	[0.52]	[0.86]	[0.39]
4+	2.20***	1.16	1.52	1.31
	[0.48]	[0.58]	[0.73]	[0.37]
**Age group** *(ref*: *15–24)*				
25–34	0.95	0.94	0.63	1.01
	[0.11]	[0.27]	[0.20]	[0.27]
35–49	0.81	0.88	0.56	0.55***
	[0.12]	[0.32]	[0.23]	[0.13]
**Education** *(ref*: *none/primary)*				
Post-Primary/Secondary	0.80**	0.72	0.72	0.94
	[0.08]	[0.24]	[0.20]	[0.27]
Tertiary/College	0.50***	0.44**	0.31***	0.58
	[0.07]	[0.18]	[0.10]	[0.21]
**Work status and remuneration** *(ref*: *no employment)*				
Paid work	1.07	0.66*	0.93	1.13
	[0.12]	[0.16]	[0.24]	[0.18]
Informal in-kind or cash paid	1.04	1.26	0.77	1.09
	[0.15]	[0.47]	[0.28]	[0.32]
**Members in the household** *(ref*: *1–3)*				
4–6	1.16	0.89	1.01	1.22
	[0.15]	[0.28]	[0.26]	[0.28]
7+	1.08	1.01	2.17**	1.50
	[0.15]	[0.34]	[0.72]	[0.37]
**Household wealth tertile** *(ref*: *lowest)*				
Middle	0.95	0.96	0.53***	0.67**
	[0.10]	[0.21]	[0.11]	[0.11]
Highest	0.59***	0.37***	0.41***	0.56***
	[0.08]	[0.10]	[0.12]	[0.11]
**Residence** *(ref*: *urban)*				
Rural residence	0.87	1.06		
	[0.12]	[0.32]		
**Constant**	0.53***	0.05***	0.43*	0.7
	[0.13]	[0.03]	[0.21]	[0.31]
**Observations**	5,978	3,510	953	1,281

Notes- Reporting odds ratio. Bolded values are statistically significant at *** p<0.01, ** p<0.05, * p<0.1; robust standard errors clustered at EA level and reported in brackets; regressions for Burkina Faso and Kenya include regional fixed effects; control variables measured at baseline (approximately 6 months prior to COVID survey); all regressions use inverse probability survey weights to account for attrition between rounds and phone ownership.

**Table 4:** Individual and household factors associated with success accessing health care during COVID-19 restrictions in Kenya, Burkina Faso, Lagos, Nigeria, and Kinshasa, DRC [LOGISTIC regression].

	Kenya	Burkina Faso	Lagos, Nigeria	Kinshasa, DRC
	Odds Ratio [Standard Error]			
**Marital status** *(ref*: *Married/In union)*				
Not Married/In Union	1.13	1.22	0.45	0.73
	[0.25]	[0.84]	[0.33]	[0.31]
**Parity** *(ref*: *nulliparous)*				
1	0.70	3.20	0.88	0.63
	[0.22]	[2.30]	[0.92]	[0.27]
2–3	0.72	1.80	0.54	0.35*
	[0.21]	[1.54]	[0.53]	[0.19]
4+	0.63	3.32	1.63	0.18**
	[0.21]	[3.41]	[1.70]	[0.12]
**Age group** *(ref*: *15–24)*				
25–34	0.90	1.41	1.32	1.30
	[0.22]	[0.86]	[1.92]	[0.57]
35–49	1.07	0.65	0.72	3.02*
	[0.30]	[0.48]	[1.09]	[1.95]
**Education** *(ref*: *none/primary)*				
Post-Primary/Secondary	1.37	1.37	5.02*	3.20**
	[0.30]	[0.89]	[4.33]	[1.87]
Tertiary/College	1.16	1.77	1.76	2.05
	[0.29]	[1.28]	[1.38]	[1.25]
**Work status and remuneration** *(ref*: *no employment)*				
Paid work	1.22	0.79	0.72	0.47*
	[0.20]	[0.25]	[0.43]	[0.20]
Informal in-kind or cash paid	1.38	0.63	0.88	2.04
	[0.37]	[0.43]	[0.94]	[1.47]
**Members in the household** *(ref*: *1–3)*				
4–6	0.95	0.94	0.69	0.90
	[0.23]	[0.51]	[0.70]	[0.61]
7+	0.82	0.34**	0.29	0.46*
	[0.22]	[0.17]	[0.34]	[0.20]
**Household wealth tertile** *(ref*: *lowest)*				
Middle	0.40***	1.26	5.19**	1.56
	[0.09]	[0.59]	[4.03]	[0.78]
Highest	0.51***	2.16	2.93*	0.70
	[0.13]	[1.19]	[1.89]	[0.32]
**Residence** *(ref*: *urban)*				
Rural residence	1.43*	1.64		
	[0.28]	[0.62]		
**Constant**	22.79***	5.55	3.82	4.52
	[11.57]	[5.87]	[7.63]	[4.16]
**Observations**	2,964	1,479	199	395

Notes- Measure of access to health care is conditional on reporting a need to access health care; Reporting odds ratios and bolded values are statistically significant at *** p<0.01, ** p<0.05, * p<0.1; robust standard errors clustered at EA level and reported in brackets; regressions for Burkina Faso and Kenya include regional fixed effects; control variables measured at baseline (approximately 6 months prior to COVID survey); all regressions use inverse probability survey weights to account for attrition between rounds and phone ownership.

The woman’s own education level at baseline had disparate associations with the outcome variables across the samples. Women with a tertiary education showed a higher risk of partial household income loss in Kenya (RRR 1.9%, 95% CI, 1.3–2.9) and Kinshasa (RRR 3.3%, 95% CI, 0.9–12.0) potentially reflecting more direct impacts of the movement restrictions on non-farm labor and wage labor [30, 36]. However, in Burkina Faso, tertiary education was associated with significantly lower risk of complete household income loss compared to no household income loss (RRR 0.2, 95% CI, 0.1–0.4). For food security and health care access education was associated with better outcomes. Controlling for other sociodemographic factors, women with tertiary education have lower odds of household food insecurity in Kenya (OR 0.5, 95% CI, 0.4–0.7) Burkina Faso (OR 0.4, 95% CI, 0.2–0.9) and Lagos (OR 0.3, 95% CI 0.2–0.6) (Table 2). Similarly, in both the urban samples, Kinshasa and Lagos, women with more than a primary education had higher odds of accessing health care when they needed it (OR 3.2, 95% CI, 1.0–10.0 and 5.0, 95% CI, 0.9–2.7, respectively) (Table 4). In the national samples (Kenya and Burkina Faso) education was not significantly associated with successfully accessing health care when the analysis controlled for other sociodemographic characteristics. However, it is worth reiterating that overall, access to needed health services was high in all geographies, ranging from 78% in Kinshasa to 94% in Lagos (Table 1).

The bivariate patterns with pre-pandemic wealth category shown in Figs 2–6 largely remained when household and individual characteristics were controlled for, with the exception of complete household income loss in Lagos. Being in the highest household wealth category pre-pandemic was associated with a lower risk of complete household income loss in Lagos (RRR 0.4, 95% CI, 0.2–0.9), but wealth was not significantly associated with the experience of partial income loss in any of the samples and, similarly, it was not significantly associated with the experience of complete household income loss anywhere aside from Lagos. (Table 2) Similarly, women from the highest wealth tertile were statistically significantly less likely to experience food insecurity in their households in all samples, and women in the middle wealth tertile were less likely to report food insecurity in both urban samples as well. (Table 3) Interestingly, there was little consistent pattern in access to health care. In Kenya, women in the middle and highest wealth tertiles had lower odds of accessing needed health care than women in the poorest households (ORs 0.4, 95% CI, 0.3–0.6, and 0.5, 95% CI, 0.3–0.8, respectively). In Lagos the pattern was reversed, with women in middle and highest wealth tertiles having higher odds of accessing needed health care (ORs 5.2,95% CI, 1.1–23.8, and 2.9 95% CI, 0.8–10.4, respectively). In Burkina Faso and Kinshasa, the relationships were non-significant (Table 4).

Finally, in the two national samples that have both urban and rural households, we find that living in a rural area is associated with lower odds of partial income loss in both Kenya (RRR 0.4, 95% CI, 0.3–0.7) and Burkina Faso (RRR 0.6, 95% CI, 0.4–0.9) and lower risk of complete income loss in Kenya (RRR 0.4, 95% CI, 0.2–0.8). (Table 2) In Kenya we find that women in rural households have higher probability of accessing needed healthcare (OR 1.4, 95% CI, 1.0–2.4), but not in Burkina Faso. (Table 4) In neither sample do we find an association between rural residence and food insecurity (Table 3).

## Discussion

In this research, we use data from four African settings to examine changes in household economic status and food insecurity, and to assess access to health care during the COVID-19 pandemic. To do so, we use rarely available representative data for women collected just prior to the COVID-19 pandemic and shortly after restrictions were put into place.

The evidence in this paper covers the first wave of the COVID-19 pandemic, in late spring and summer of 2020. At that time, many governments in Africa put in place restrictive lockdown measures to contain the epidemic, while acknowledging that these measures would have secondary economic and social effects. Evidence from the early wave of the pandemic suggested that the direct morbidity and mortality impacts of the first wave of the pandemic were comparatively limited in Africa, likely due, in part, to the lockdowns [1, 15]. At the time of this writing, however, the Delta variant is fueling a new wave of infections across the African continent [10]. As access to vaccines is still extremely limited, policy makers are once more making difficult decisions about implementing lockdowns. This analysis focuses on documenting the level and distribution of several negative socioeconomic outcomes during the COVID-19 lockdowns. Our data are only descriptive. We do not infer direct causal links between the COVID-19 restrictions and the outcomes described here. Even among these four samples, there are differences in government policy, COVID-related outreach, agricultural seasonality, health and economic infrastructure, levels of conflict, baseline wealth, and infection rates. All of these unobserved factors, and many others, could mediate the impact of COVID-19 restrictions in unpredictable ways. Nevertheless, even without perfect understanding of the causal pathways, documenting where the secondary effects of lockdowns were observed in the first wave can help governments to respond effectively to mitigate the overall social and economic costs of future restrictions.

Our data reflect a considerable economic contraction during the first wave the pandemic. Similar to other analyses in African countries [24, 27], we find that the scale of household income loss is substantial, with over 90% of women reporting that their households lost some or all of their income during the COVID-19 restrictions in three of our four geographies. The pattern of income loss diverges from those recorded in the early stage of the pandemic in Europe and the USA, where poorer households bore the brunt of the economic losses [17, 38]. In three of our geographies, the economic shock was distributed similarly across the pre-pandemic wealth spectrum. This aligns with similar findings from national studies in Malawi, Nigeria, Ethiopia, Uganda and South Africa [24, 27, 29, 31]. However, it diverges from findings in urban Ethiopia, [28] rural Uganda [30] and in Nigeria [39]. Although Lagos, Nigeria is an exception in our data as well, showing a clear wealth gradient in complete income loss.

In our two national samples of women aged 15–49, we found that rural households appear to have experienced lower levels of income loss once household wealth and other sociodemographic characteristics are controlled for. While we cannot comment on the causal mechanism here, and we do not have measures of sources of household income, this does align with prior work suggesting that rural agricultural areas may have experienced less income shock during the first wave of the pandemic due to lower levels of integration into the non-farm economy [30, 40]. It bears repeating that this data was collected within the first wave of the pandemic and it is possible that the patterns described here have evolved, but in the initial wave of COVID-19 the household economic contraction appears to be both deep and wide, and occurring largely in the absence of large-scale government income support programs.

Early in the pandemic, the World Food Programme estimated that the number of people facing food insecurity globally in 2020 would likely nearly double from 135 million to 265 million [41]. We find high levels of food insecurity in all four of our geographies. Although we do not have pre-pandemic measures of food insecurity in our study households, the majority of respondents who reported household food insecurity also indicated that it had increased during the COVID restrictions, and the measures of prevalence are high compared to pre-pandemic national measures. Aside from the immediate effect of household income loss, COVID-19 is disrupting global supply chains, which cuts into both the availability of many foods, and the ability of food producers in Africa to access global markets. Simultaneously, restrictions on movement and gatherings can curb the local and informal food markets upon which many households rely. These multiple threats to the food supply are threatening to generate a nutritional health crisis, even where the worst effects of the COVID-19 pandemic have been avoided [42, 43]. As in many economic crises, our multivariate analyses show a pattern of overlapping vulnerabilities, where women who have lower education and lower household wealth have a higher odds of food insecurity. Moreover, in three of our studied geographies, women with children appear to be at higher risk of experiencing food insecurity than those without. Although our study does not directly evaluate children, the implication is that a significant percentage of children will suffer food insecurity as a result of the pandemic.

Finally, our findings on health care access are a relative bright spot in the data. By and large, the women in our study did not report that they struggled to access health care. Prior to this work, we anticipated that a major area of disruption would be health care. We predicted health care systems could become stressed by the pandemic, and women would be hampered by restrictions on movement, economic constraints, and concerns about being exposed to COVID-19 in health settings. But we largely found that the women across the wealth spectrum were able to access health care when they needed it. As this analysis is conditional on the respondent needing health care, it’s possible that this finding is affected by selectivity if there are meaningful sociodemographic differences in the percentages of women who need health care. But we also did not find a wealth gradient or urban-rural difference among women reporting needing health care. As we do not have pre-pandemic measures of need for health care, it is possible that respondents have differentially adjusted their evaluation of ‘need’ in the COVID context. But we did not find evidence of women attempting and failing to access health care at this stage of the pandemic.

Our study does have several limitations. The follow-up survey was conducted as a phone survey due to the risks of face-to-face interviewing during COVID-19; although we have used post stratification weights to limit the impact of differential response rates by phone ownership, there remains the possibility of selection bias in our results. Second, our findings are descriptive only. Although we do have a household wealth measure at baseline, we did not have measures of food insecurity or household income in our baseline study, as it was designed as a study of family planning rather than household economics. We are unable to independently measure change in food security or health care access compared to pre-COVID. We rely on women’s subjective evaluations of their own household income, food insecurity and health care needs during the COVID restrictions. In addition, although the COVID-19 restrictions were in place for similar lengths of time in each geography, our interviews began during the restrictions in two geographies, and approximately 2–3 weeks after they had been fully or partially lifted in two others. It is hard to anticipate how exactly that might impact respondent reports or whether any recovery had taken place already in those places where restrictions had been lifted. Finally, the patterns and impacts captured here reflect only the immediate impact of the global economic shock of COVID-19. Since this data was collected, there have been considerable changes in the trajectory of the pandemic globally, including the development of vaccines, and the advent of the Delta variant. Further follow up with these households is currently taking place to examine whether the socioeconomic impacts documented here persist as the pandemic evolves.

## Conclusions

Although descriptive in nature, the findings reported here support some of the concerns of the global community about the secondary effects of COVID-19 lockdowns in Africa. Food insecurity, in particular, is increasing and concentrated among already vulnerable households. But we also find some patterns contrary to a priori expectations. We find little evidence of population level failure of health systems or barriers to health care access. And, contrary to patterns observed in HICs, the income shock of COVID-19 appears to be distributed similarly across wealth categories in three of our four samples. Consequently, policy efforts at general economic relief may be best designed at the population level, rather than targeted; whereas there is an urgent need to focus on food support for the poorest households.

## Supporting information

S1 Appendix PMA COVID-19 questionnaire. (PDF)

S2 Appendix PMA COVID log files. (LOG)

S1 Data PMA COVID-19 pooled data. (ZIP)

## References

[1] UNDP Regional Bureau for Africa. Long Term Socioeconomic Impacts of COVID-19 Across diverse African Contexts. 2021 March 11 [Cited 2021 August 21] https://www.africa.undp.org/content/rba/en/home/library/reports/analysing-long-term-socio-economic-impacts-of-covid-19-across-di.html

[2] Madden, P. Figures of the week: The macroeconomic impact of COVID19 in Africa. 2020 April 16 [Cited 2021 August 21] https://www.brookings.edu/blog/africa-in-focus/2020/04/16/figures-of-the-week-the-macroeconomic-impact-of-covid-19-in-africa/

[3] Gondwe, G., Assessing the Impact of COVID-19 on Africa’s Economic Development. 2020 July. [Cited 2021 August 21] https://unctad.org/system/files/official-document/aldcmisc2020d3_en.pdf

[4] Adams-Prassl A, Boneva T, Golin M, Rauh C. Inequality in the Impact of the Coronavirus Shock: Evidence from Real Time Surveys Journal of Public Economics. 2020; 189:1–33

[5] Madgavkar A, White O, Krishnan M, Mahajan D, Azcue X. COVID-19 and Gender Equality: Countering the Regressive Effects. Mckinsey Global Institute. July 15, 2020. [Accessed August 20, 2020.] https://www.mckinsey.com/featured-insights/future-of-work/covid-19-and-gender-equality-countering-the-regressive-effects#

[6] Roberton T, Carter E, Chou V, et al. Early estimates of the indirect effects of the COVID-19 pandemic on maternal and child mortality in low-income and middle-income countries: a modelling study. Lancet Glob Health. 2020; 8(7):e901–e908. doi: 10.1016/S2214-109X(20)30229-1 32405459 PMC7217645

[7] Hodgins S, Saad A. Will the Higher-Income Country Blueprint for COVID-19 Work in Low-and Lower Middle-Income Countries? Glob Health Sci Pract. 2020; 8(2): 136–143. doi: 10.9745/GHSP-D-20-00217 32522765 PMC7326511

[8] Teachout M, Zipfel C. The economic impacts of COVID-19 lockdowns in sub-Saharan Africa. International Growth Centre Policy Brief, May 2020 [Cited August 20, 2020] https://www.theigc.org/wp-content/uploads/2020/05/Teachout-and-Zipfel-2020-policy-brief-.pdf

[9] Lawal Y. Africa’s low COVID-19 Mortality rate: A paradox? Int J Infect Dis. 2021; 102: 118–122 doi: 10.1016/j.ijid.2020.10.038 33075535 PMC7566670

[10] Njenga M, Dawa J, Nanyingi M, Gachohi J, Ngere I., Letko M, et al. Why is There Low Morbidity and Mortality of COVID-19 in Africa? Am J Trop Med Hyg. 2020; 103(2): 564–569. doi: 10.4269/ajtmh.20-0474 32484156 PMC7410455

[11] El Sadr W, Justman J. Africa in the Path of COVID-19. N Eng J Med. 2020; 383:e11. doi: 10.1056/NEJMp2008193 32302075

[12] Lone A, Ahmad A. COVID-19 Pandemic- an African Perspective. Emerg Microbes Infect. 2020; 9(1):1300–1308. doi: 10.1080/22221751.2020.1775132 32458760 PMC7473237

[13] Ataguba J. COVID-19 pandemic, a war to be won: understanding its economic implications for Africa. Appl Econ Health Policy. 2020; 18:325–328. doi: 10.1007/s40258-020-00580-x 32249362 PMC7130452

[14] Aizenman, N. The Downs and Ups of Africa’s current COVID Surge. 2021 July [Cited 2021 August 21] https://www.npr.org/sections/goatsandsoda/2021/07/23/1019281078/the-downs-and-ups-of-africas-current-covid-surge

[15] The World Bank. Amid Recession, sub-Saharan Africa Poised for Recovery. 2021 March 31. [Cited 2021 August 21] https://www.worldbank.org/en/news/press-release/2021/03/31/amid-recession-sub-saharan-africa-poised-for-recovery

[16] Mwananyanda L, MacLeod W, Kwenda G, et al. COVID-19 deaths in Africa: prospective systematic post-mortem surveillance study. Br Med J.2021; (372): n334. 33597166 10.1136/bmj.n334 PMC7887952

[17] Maeda J, Nkengasong J. The Puzzle of COVID-19 In Africa. Science. 2021; 371:6524; 27–28. doi: 10.1126/science.abf8832 33384364

[18] Ondoa P, Kebede Y, Loembe M, Bhiman J, Tessema S, Sow A, et al. COVID-19 testing in Africa: lessons learnt. Lancet Microbe, 2020;1(3):e103–e104. doi: 10.1016/S2666-5247(20)30068-9 32835338 PMC7333988

[19] Mbow M, Lell B, Jochems S, Cisse B, Mboup S, Dewals B, et al. COVID-19 in Africa: Dampening the Storm? Science. 2020; 369(6524): 624–626. doi: 10.1126/science.abd3902 32764055

[20] Fengler, W, Nelly MF, Gill I, Baduel B. South Africa After COVID-19-light at the end of a very long tunnel. 2021 July 13. [Cited 2021 August 20] https://www.brookings.edu/blog/future-development/2021/07/13/south-africa-after-covid-19-light-at-the-end-of-a-very-long-tunnel/

[21] Nordling L. Africa’s pandemic puzzle: why so few cases and deaths? Science. 2020; 369(6524): 756–757. doi: 10.1126/science.369.6505.756 32792376

[22] Zwedu S. Africa: In the Fight Against Covid-19, The Unsung Continent. Bill and Melinda Gates Foundation (2020) [Accessed Sept 1, 2020] https://www.gatesfoundation.org/TheOptimist/Articles/coronavirus-solomon-zewdu-africa-response

[23] Diop BZ, Ngom M, Pougué Biyong C, et al The relatively young and rural population may limit the spread and severity of COVID-19 in Africa: a modelling study BMJ Glob Health 2020;5:e002699. doi: 10.1136/bmjgh-2020-002699 32451367 PMC7252974

[24] Egger D, Miguel E, Warren S, Shenoy A, Collins E, Karlan D, et al. Falling living standards during the COVID-19 crisis: Quantitative evidence from nine developing countries. Sci. Adv. 2021; 7(6): 1–12. doi: 10.1126/sciadv.abe0997 33547077 PMC7864564

[25] The World Bank. COVID-19 (Coronavirus) Response in Africa. [Cited 2021 August 21] https://www.worldbank.org/en/region/afr/coronavirus.

[26] Africa Center for Strategic Studies. Food Insecurity Crisis Mounting in Africa. 2021 February [Cited 2021 August 21] https://africacenter.org/spotlight/food-insecurity-crisis-mounting-africa/

[27] Josephson A., Kilic T., & Michler J. D. Socioeconomic impacts of COVID-19 in low-income countries. Nat Hum Behav. 2021; 1–9. 33785897 10.1038/s41562-021-01096-7

[28] Abate, G T.; de Brauw, A; and Hirvonen, K. Food and nutrition security in Addis Ababa, Ethiopia during COVID-19 pandemic: June 2020 ESSP Working Paper 145. Washington, DC: International Food Policy Research Institute (IFPRI). [Cited 2021 August 20] 10.2499/p15738coll2.133766

[29] Adjognon S.G., Bloem J. Sanoh A. The coronavirus pandemic and food security: Evidence from West Africa, Food Policy, 2021 April. 10.1016/j.foodpol.2021.102050 PMC9758592 36570061

[30] Mahmud M. and Riley E. Household response to an extreme shock: Evidence on the immediate impact of the COVID-19 lockdown on economic outcomes and well-being in rural Uganda. World Dev. 2020 December 10.1016/j.worlddev.2020.105318 PMC8446716 34548741

[31] Visagie J. Turok I. Rural–urban inequalities amplified by COVID-19: evidence from South Africa, Area Development and Policy. 2021; 6:1, 50–62

[32] PMA. Covid Survey Weights Construction Memo [Cited 2021 August 21] https://www.pmadata.org/sites/default/files/2020-07/COVID%20Survey%20Weight%20Construction%20Memo.pdf.

[33] Filmer D., & Pritchett L. H. Estimating wealth effects without expenditure data—or tears: an application to educational enrollments in states of India. Demography, 2001; 38(1): 115–132. doi: 10.1353/dem.2001.0003 11227840

[34] Food and Agriculture Organization of the United Nations. FAOSTAT; 2021 [Cited 2021 August 20] http://www.fao.org/faostat/en/?#data/FS

[35] Beaubien, J. Locust swarms threaten parts of East Africa. 2021 Jan 21. [Cited 2021 August 26] https://www.npr.org/2021/01/19/958543535/locust-swarms-threaten-parts-of-east-africa

[36] Swinnen J, ed.; and McDermott J, ed. COVID-19 and global food security. Washington, DC: International Food Policy Research Institute (IFPRI). 2020. [Cited 2021 August 21] 10.2499/p15738coll2.133762

[37] Thurlow J. COVID-19 lockdowns have imposed substantial economic costs on countries in Africa. In COVID-19 and global food security, eds. Swinnen Johan and McDermott John. Part One: Food security, poverty, and inequality, Chapter 4, Pp. 23–25. Washington, DC: International Food Policy Research Institute (IFPRI). 2020 [Cited 2021 August 21] 10.2499/p15738coll2.133762_04

[38] Blundell R, Costa Dias M, Joyce R, Xu X. COVID-19 and Inequalities. Fisc Stud. 2020; 41: 291–319. doi: 10.1111/1475-5890.12232 32836542 PMC7362053

[39] Amare M., Abay K. Tiberti L., and Chamberlin J. Impacts of COVID-19 on Food Security: Panel Data Evidence from Nigeria,” Food Secur. 2021. 10.1016/j.foodpol.2021.102099 PMC9758590 36570064

[40] Aggarwal, S., Jeong, D., Kumar, N., Robinson, J. and Spearot, S. (2020) Did COVID-19 market disruptions disrupt food security? Evidence from households in rural Liberia and Malawi,” NBER Working Paper 2020 October [Cited 2021 August 21] https://www.nber.org/papers/w27932

[41] World Food Programme. COVID 19 will double the number of people facing food crises unless swift action is taken. 2020 April 21 [Cited August 15, 2020] https://www.wfp.org/news/covid-19-will-double-number-people-facing-food-crises-unless-swift-action-taken

[42] Bene C. The resilience of local food systems and links to food security- A. review of some important concepts in the context of COVID-19 and other shocks. Food Secur. 2020; 12: 805–822. doi: 10.1007/s12571-020-01076-1 32837646 PMC7351643

[43] Deveraux S, Bene C, Hoddinott J. Conceptualizing COVID-19s impacts on household food security Food Secur. 2020; 12: 769–772. 10.1007/s12571-020-01085-0 PMC7358330 32837651

